# Development of a Hybrid Method to Generate Gravito-Inertial Cues for Motion Platforms in Highly Immersive Environments

**DOI:** 10.3390/s21238079

**Published:** 2021-12-02

**Authors:** Jose V. Riera, Sergio Casas, Marcos Fernández, Francisco Alonso, Sergio A. Useche

**Affiliations:** 1 Institute of Robotics, Information Technologies and Communication Research (IRTIC), University of Valencia, 46980 Valencia, Spain; j.vicente.riera@uv.es (J.V.R.); sergio.casas@uv.es (S.C.); marcos.fernandez@uv.es (M.F.); 2 Faculty of Psychology, University of Valencia, 46010 Valencia, Spain; francisco.alonso@uv.es; 3 Research Institute on Traffic and Road Safety (INTRAS), University of Valencia, 46022 Valencia, Spain

**Keywords:** hybrid gravito-inertial cues, motion cueing algorithms, motion platform, Virtual Reality (VR), simulators, road environments

## Abstract

Motion platforms have been widely used in Virtual Reality (VR) systems for decades to simulate motion in virtual environments, and they have several applications in emerging fields such as driving assistance systems, vehicle automation and road risk management. Currently, the development of new VR immersive systems faces unique challenges to respond to the user’s requirements, such as introducing high-resolution 360° panoramic images and videos. With this type of visual information, it is much more complicated to apply the traditional methods of generating motion cues, since it is generally not possible to calculate the necessary corresponding motion properties that are needed to feed the motion cueing algorithms. For this reason, this paper aims to present a new method for generating non-real-time gravito-inertial cues with motion platforms using a system fed both with computer-generated—simulation-based—images and video imagery. It is a hybrid method where part of the gravito-inertial cues—those with acceleration information—are generated using a classical approach through the application of physical modeling in a VR scene utilizing washout filters, and part of the gravito-inertial cues—the ones coming from recorded images and video, without acceleration information—were generated ad hoc in a semi-manual way. The resulting motion cues generated were further modified according to the contributions of different experts based on a successive approximation—Wideband Delphi-inspired—method. The subjective evaluation of the proposed method showed that the motion signals refined with this method were significantly better than the original non-refined ones in terms of user perception. The final system, developed as part of an international road safety education campaign, could be useful for developing further VR-based applications for key fields such as driving assistance, vehicle automation and road crash prevention.

## 1. Introduction

Virtual Reality (VR) is beginning to impact our day-to-day life, with a growing number of potential applications on key fields such as vehicle automation, road users’ training and traffic safety management. Only forty years ago, it was impossible to think that people would be able to teleport to a completely different virtual space just using a pair of glasses. Fortunately, thanks to recent hardware and software advances in computer graphics, this is a reality today. Of course, advancements in computer graphics should come alongside the rest of VR elements [1,2].

For this reason, nowadays, motion generation is being applied to a larger and larger number of applications, mainly thanks to the increasing availability of motion hardware. The purpose of these motion-based systems is to transfer to the VR application the same motion sensations that would be experienced in the real world by the users [3]. These motion systems are based on a series of actuators that move following the commands generated by a computer. These systems are generally known as motion platforms and are commonly used in vehicle simulators. 

The main goal of motion platforms is to reproduce the accelerations that the VR users should feel, but always taking into account the physical limits of the actuators [1,2,4]. However, the motion envelope of these robotic mechanisms is often much more limited than the real motion of the simulated object.

**Figure 1:**
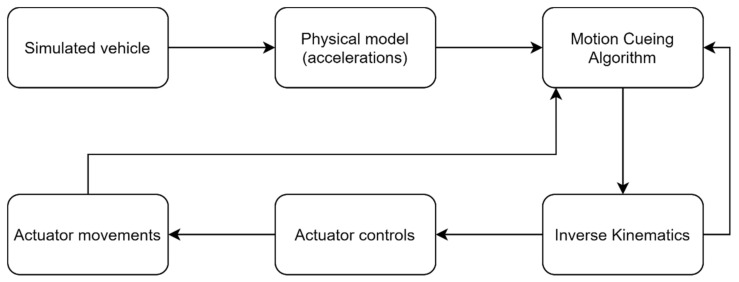
Classical data-flow scheme for motion cue generation in a motion-based simulator.

For this reason, when using motion platforms in a real-time simulation, it is impossible to reproduce the full extent of the accelerations that the simulated vehicle is suffering. To overcome this problem, a classical approach consists on filtering the input accelerations by means of so-called washout filters [3,4,5,6,7]. Washout-based filtering is the most common way to implement a Motion Cueing Algorithm (MCA). An MCA is an algorithm that calculates the appropriate motion commands for recreating gravito-inertial cues. The movements generated by the MCA go to the inverse kinematics layer, which transforms them into actuators’ positions to ultimately generate real movement through the actuators’ control. Figure 1 shows the most common process used in motion cueing generation. 

As it can be noticed, in a classical approach, the simulated vehicle provides the necessary inputs to the process. The physical state of this simulated vehicle—usually its acceleration and angular velocity—is calculated by the VR application to provide proper visual motion, but it is also used to feed the washout filters so that motion cues are generated [3,5]. However, what happens if we want to move the motion platform according to a flow of images where we do not have a simulated vehicle? What if we want to combine computer-generated images—with physical information and recorded imagery—without it? This is the question this paper tries to answer.

The development of new capture devices and new ways to reproduce VR scenes makes the classical way to generate motion cues for motion platforms inapplicable if they lack physical information, as it usually occurs with 360° videos. A similar situation occurs in Augmented Reality (AR) scenes, where virtual objects and real images blend [6,8]. In this case, the accelerations of the virtual objects could be easily calculated by applying a physics-based model. Notwithstanding, if we want to move the motion platform according to real images, we need to identify alternative solutions [2,9].

In a first approximation, this can be seen as a visual tracking problem, solved, for instance, through spatio-temporal video segmentation, but this is not always the case. On most VR applications, the visual information is presented in a first-person view [4,5,6,9]. In these cases, it is not enough to perform the tracking of an object because what we want is to calculate the accelerations of the camera, which represents the view of the user [8,9]. The motion cues should match the visual motion, but without proper inputs, the MCA cannot work.

On the other hand, another possible solution to this problem can be the installation of accelerometers in the object whose motion needs to be extracted. Although this seems perfectly applicable, there are a number of drawbacks. First, there is an economic impact on the project. Second, some images may be already recorded and, therefore, this solution may be inapplicable. Finally, most cameras used for these purposes incorporate hardware image stabilizers. Thus, the accelerations that the object is suffering would not be faithfully represented in the obtained image, and this could have negative repercussions for the users’ perception. 

Given all these problems, this paper aims to present a method that allows defining inertial cues for motion platforms in cases where all or some of the physical information that corresponds to the visual motion information is not present, as occurs with images recorded with video cameras in the real world.

Our method is hybrid since it deals with the combination of classical motion cues—generated from simulated vehicles—and *non-classical* motion cues—generated from pre-recorded imagery. In order to test the proposed method, a highly immersive non-real-time system was used, based on an immersive cylindrical stereoscopic-projected screen with a 12-seat motion platform, where the visual flow is divided into scenes generated by computer graphics and real pre-recorded video scenes. In the former, the motion cues come from the physical model that is being simulated in the virtual world. In the second, the motion cues come from the result of the application of the presented method.

The combination of classical and non-classical motion cues was possible thanks to various tools developed by the authors and the use of the Wideband Delphi-based tuning method that will be explained later. To the best of our knowledge, the application of this consensus-based method to motion platforms has not previously been described in the academic literature.

The rest of the paper is organized as follows: Section 2 presents a short review of the state of the art of motion platforms and motion cueing algorithms. Then, Section 3 provides a presentation of all the elements used to carry out the experiments, making a complete description of the system, focusing on the motion platform and the different experiments carried out with experts and users. Section 4 deals with the results. Finally, Section 5 appends a synthesis of the study outcomes and raises some key issues to be considered in future work.

## 2. Related Work

Most VR applications use visual and acoustic information to generate a sense of immersion in their users. Although it is widely accepted that most of the perceptual information that the human brain receives comes through these two senses, it is also true that, in order to achieve a complete and highly immersive virtual experience, other perceptual cues need to be included in the system. 

VR applications that deal with physics-based motion need to find a way to include motion cues in the system. This is usually accomplished by means of an electromechanical motion platform and an MCA. The first MCA was designed for flight simulators, and the Stewart–Gough design [1] has been the preferred solution for this kind of application ever since motion cues were added to VR applications. This 6-Degree of Freedom (DOF) parallel solution, also known as hexapod, is almost a de facto solution for flight simulation and driving simulation. However, there are other alternatives. In fact, in recent years, several reduced-DOF solutions have been proposed with success. 2-DOF and 3-DOF parallel setups are fairly common as a low-cost alternative for motion simulation [2,3,4,5,6]. 4-DOF and 5-DOF parallel systems [7,8,9] are possible but less common since their complexity and cost are similar to a 6-DOF solution, and therefore, they do not usually represent a low-cost alternative. 1-DOF systems are extremely rare and only used for very specific situations. 

The problem with parallel motion platforms is that the workspace is usually very much reduced because of the interdependence between the different kinematic chains. However, the parallel design allows for great payloads with relatively reduced electrical power. On the contrary, serial motion devices, such as [10], are usually a much more expensive solution, but they provide better motion workspaces and a great deal of flexibility for motion software. Therefore, they can be considered an alternative for the hexapod, but not a low-cost solution, since they require powerful, expensive engines. 

Regarding the motion algorithms, the first studies about motion cueing generation in VR applications were published by Schmidt and Conrad in the late 1960s and early 1970s [11,12,13] as part of their NASA work. However, the most credited research is the work performed by the University of Toronto Institute for Aerospace Studies (UTIAS) researchers [14,15,16], who studied in detail the so-called classical washout algorithm. This algorithm has been used in countless applications with different motion platforms and objectives [3,17,18,19]. 

Nevertheless, several other solutions have been proposed to improve some of the disadvantages of this algorithm. Parrish et al. proposed a modified version of the classical washout [20] that is known as the adaptive algorithm. This algorithm tries to exploit the workspace of the motion platform in a more efficient way since the parameters of the classical washout have to be set up for a worst-case scenario to ensure that the limits of the motion device are not reached. A few years later, the optimal algorithm was proposed in an attempt to obtain a motion cueing algorithm that can be considered optimal with respect to control theory [21,22]. This solution includes a model of human perception, but its optimality is disputed since it is only optimal for the perception model and hypothesized conditions established in the mathematical derivation of the algorithm.

Recently, Dagdelen et al. have proposed an MCA based on model-based predictive control [23]. This approach has received a lot of attention lately [24,25,26,27], since the classical, the adaptive and the optimal algorithms have been used for more than thirty years, without any of them having proven to be considerably superior to the others. In fact, the classical algorithm is still the most utilized one because it is simple, effective and relatively easy to implement, even though still limited.

All these algorithms are applied in real-time using the physical state of the entity whose motion needs to be simulated. However, they can also be applied with non-real-time inputs. This may be the case of rollercoasters, some train simulators or VR applications, such as haptic devices [28] that do not use real-time physics simulation but have the possibility of calculating the motion state of the simulated entity and therefore, apply an MCA as if they were running in real-time. In this case, the output motion could be studied and refined to suit the user’s needs, which represents an important advantage in comparison to systems that obtain the physical state input in real-time. 

One of the problems of MCAs is that they lack a standard mechanism to compare and assess them. Therefore, it is difficult to assert that an algorithm is the best one. Since the problem is subjective, the most common evaluation is based on questionnaires with different groups of pilots/drivers. Nevertheless, there has been a recent interest in appraising the motion generation by means of objective assessment methods [28,29]. However, there is still much work to do to obtain a standardized objective assessment system. 

Another important issue is how to optimally tune these algorithms so that their performance is as good as it can be. This aspect is crucial because these algorithms usually have a great deal of parameters that modify the behavior of the motion platform. These parameters are used to reach a suitable trade-off between the motion fidelity and the compliance with the motion platform’s physical limits. Unfortunately, the use of subjective assessment methods has caused the tuning of these algorithms to be less studied since the procedure is often not systematic. One of the earliest and few studies about this topic was conducted by Grant [30], who proposed an expert system to help tune washout algorithms [31]. Grant chose not to use objective assessment methods, despite the fact that they allow assessing the performance of the MCA in a more systematic way [32]. Nevertheless, the difficulty of obtaining a proper objective function to measure the suitability of the motion generation makes them difficult to apply in some situations. 

However, motion is not the only extra-audiovisual cue that needs to be addressed in VR applications. Other perceptual cues like smell [33], depth or touch may be included too, creating truly multimodal applications that might increase the users’ immersion. Yet, this kind of multimodal setup is still rare, and few works can be reported in the literature. One of the earliest multimodal devices is Sensorama [34]. This device allowed a person to watch a stereoscopic film enhanced with stereo sound, wind, aromas, seat motion and vibration. However, the system only showed a film without virtual content, and therefore its consideration as a VR system is arguable. This enhanced cinema concept is also present in some commercial solutions that are sometimes marketed as 4D or 5D cinema [35,36]. Some of these solutions offer stereoscopic films alongside pre-recorded motion. Yet, to the best of our knowledge, these devices and the algorithms that control them have not been presented to the academic community.

## 3. Materials and Methods

### 3.1. System Description

**Figure 2:**
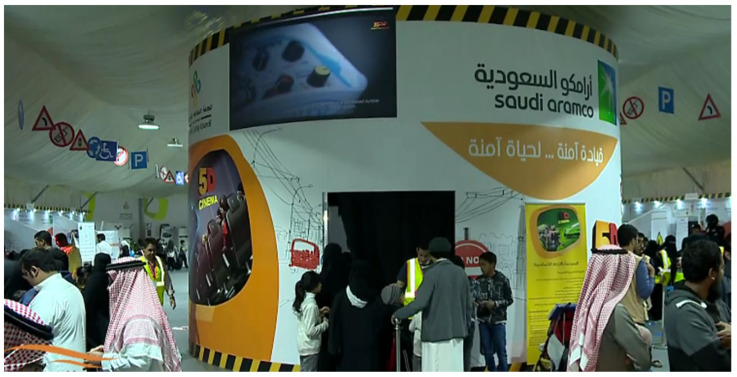
The external cylinder of the immersive theater (referred to as 5D cinema for marketing reasons).

**Figure 3:**
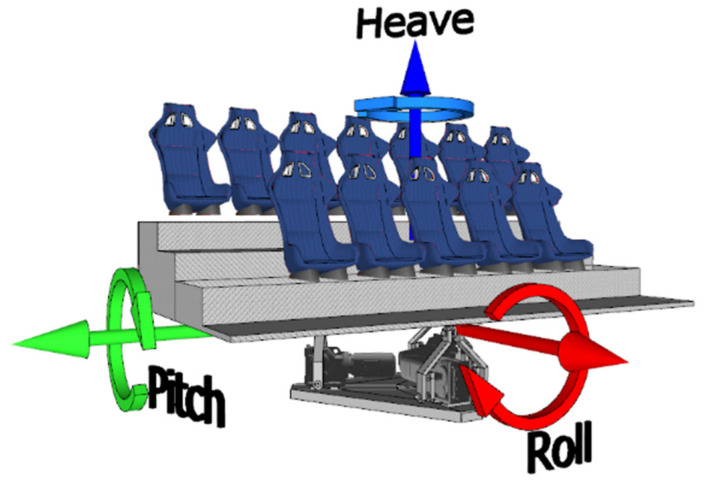
Design and degrees of freedom of the T1R2 3-DOF motion platform.

Before describing the proposed method, it is important to describe the system for which the method was conceptualized and assessed. The system features a VR-based application developed from scratch as part of an educational road safety campaign within the scope of a traffic safety program in the Kingdom of Saudi Arabia (KSA). The system consists of a cylindrical 5D theater with a total projected screen of 33 square meters in a 7-m diameter cylinder with a horizontal field of view of 180°. In the center of the cylinder, a 3-DOF motion platform was placed. The motion platform was designed for 12 users distributed in two rows. Figure 2 and Figure 3 show the system and the motion base.

The complete feature list of the system includes:

- A 450″ screen.
- A 7.1 THX audio system, including a microphone system.
- 2 x 2K HD 5500 lumens DLP projectors.
- A blending and warping system.
- A full HD video camera used for some Augmented Reality scenes.
- A computer with an Intel Xeon processor with 16 GB of RAM memory.
- A pair of active stereo glasses.
- A smoke machine.
- A smell machine.
- 12 Samsung Galaxy S7 tablets—one for each user—to make the film interactive.
- 12 water and 12 air valves to include air and water feedback for each user.

### 3.2. The Motion Platform

As previously mentioned, a 3-DOF parallel motion platform was designed and built ad hoc for this application. This robotic mechanism uses three electrical engines to generate rotational and translational movements for the 12 users of the software application. This parallel device provides translational vertical motion—denoted as *heave*—and two types of rotational motion—frontal and lateral, denoted as *pitch* and *roll* respectively—that allows the mechanism to tilt around the longitudinal and lateral axes, respectively, as seen in Figure 3. As can be seen, it is a T1R2 (one translational, two rotational) parallel motion platform.

**Figure 4:**
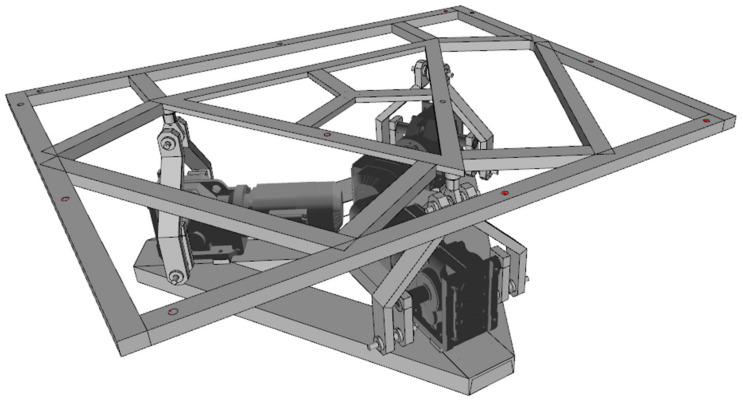
Motor placement and base design.

**Figure 5:**
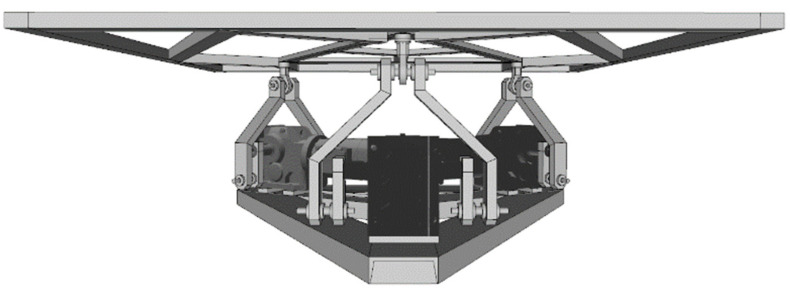
Frontal view of the motion platform design.

The engines are arranged as seen in Figure 4 and Figure 5. With this configuration, we can use the 3-DOF inverse kinematics formulation derived in [37], since this device uses the same kinematic chains, although with a reinforced mechanical structure to hold and move the weight of 12 people.

In Table 1, the main features describing the T1R2 3-DOF parallel mechanism are displayed. As can be seen, the motion envelope of the motion platform is small. However, it is powerful enough to create large linear and angular accelerations.

In addition to the design, the selected motors are an essential aspect to achieve relatively large accelerations. It is very important that the device reach high accelerations so that the software can introduce certain effects according to the users’ requests. Otherwise, there might be a need for short, high-frequency movements, and the motors would not be able to mechanically reproduce them. The device was designed to move up to 1500 kg (100 kg per user with 12 users, plus 300 kg for the structure, seats, tablet, etc.). Therefore, powerful motors were needed. 

In order to meet these requirements, three 2.2 kW SEW-EuroDrive motors were installed. These motors included a gearbox with a reduction of 58.34. Their nominal speed was of 1400 rpm, and the nominal output torque was of 880 N·m. They were also equipped with built-in brakes and high-precision encoders. The connecting rod incorporated into the motor shaft—the one that acts directly on the motion platform—was 12 cm between axles. This is mechanically equivalent to the connected rod described in [37].

Regarding the limits of the movements of the platform, these are given by the constructive design and are loaded in the kinematics configuration files, which is the software layer that indicates to each motor the specific position it must adopt to achieve a certain combination of degrees of freedom.

Once the platform workspace is known, another problem is the combination of different degrees of freedom. This means that, if, for example, the platform is requested to be at its maximum height, the pitch and roll that can be provided at that moment are zero. In our case, the strategy used to reach a consensus between the requested degrees of freedom consists on scaling down each degree of freedom, based proportionally on both what is requested and the maximum available.

### 3.3. The Film and the Users’ Experience

As previously mentioned, the motion-based system was part of an edutainment road safety campaign [38]. The 5D theater was one of the main activities. The goal of this 5D interactive theater was to show different road safety scenarios to 12 users, using Mixed Reality. These scenarios included accidents and potentially dangerous situations. For that purpose, the application consisted of a 10-min interactive film. 

The film was created using both 3D computer-generated virtual scenes and pre-recorded video with real images. The film was divided into 14 sections, each one having a particular educational content to transmit to the users. Some sections used real images obtained with real cameras and other sections used computer-generated 3D scenes.

Some scenes are computer-generated because they recreate accidents and it is not desirable to cause real accidents to show them. Other scenes are recorded because they show dangerous/defective roads or show examples of real reckless behavior recorded on video.

The 3D scenes consisted of animations created with Unity 3D. A part of the city of Dammam, KSA, was modeled and included in the application. In addition, people, traffic, and vehicles—with their corresponding physics model—were also included. In order to increase the sense of immersion, a first-person perspective was employed to display the virtual content.

**Figure 6:**
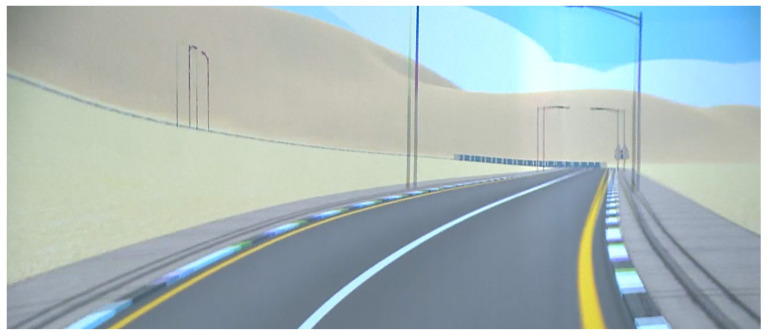
Screenshot from the graphic generated image.

**Figure 7:**
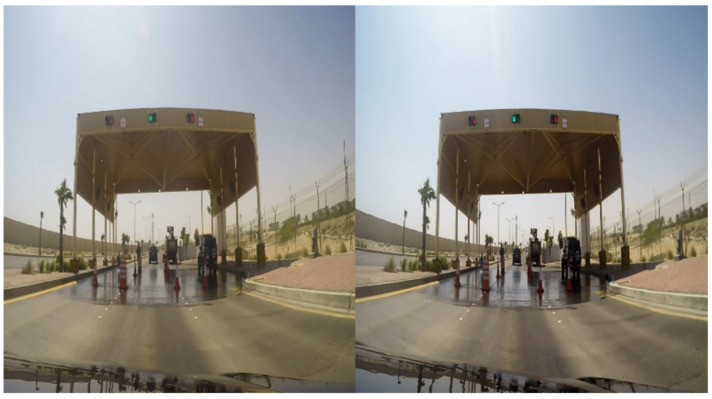
Screenshot of a real stereoscopic pair captured with the recording device.

**Figure 8:**
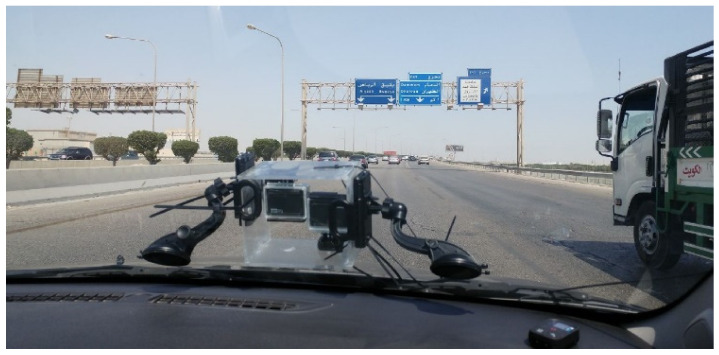
Recording devices installed in a car while filming.

The real video scenes were recorded by means of two 4K GoPro cameras. These cameras were placed in a real car and captured videos of the city of Dammam. These images were later edited and adapted to the different parts of the film where it was convenient to give a 100% realistic vision of different risks or dangerous attitudes while driving. Figure 6 and Figure 7 show a sample of a computer-generated virtual image and a real image, respectively. Figure 8 shows the GoPro cameras, used to record real images, installed in a car.

**Figure 9:**
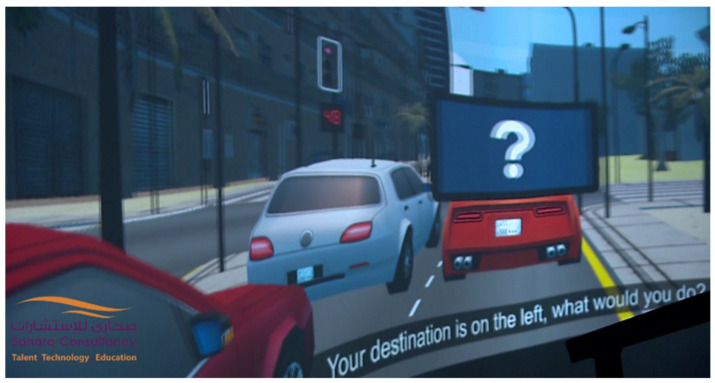
Projected screen in a virtual scene of the film with a question for the users.

During the film, questions were asked to the users using the tablets installed near the seats. To do so, the simulation server of the movie was in communication with the users’ tablets. According to the answers, the system behaved differently. If the majority of the responses were correct, it was understood that the concept to be taught in that part of the film was learned. Therefore, there was no need for any especially abrupt system behavior to catch the users’ attention. On the contrary, if most of the responses were wrong, the system behaved somewhat more abruptly, trying to make users aware of their mistakes. Figure 9 shows an example of a question asked to the users. The question appears on the theater screen, and a virtual tablet is shown to prompt users to respond to the question on their own tablet.

In addition, when a specific user failed a question, a valve controlled by the simulation server was activated, throwing some vaporized water to the user in order to make them briefly uncomfortable and aware of the failure. Similarly, when the answer was correct, the user received a gentle air blow (through another valve), generating a pleasant sensation.

Moreover, depending on the content being represented by the scene, a smell machine released different scents throughout the theater to increase the sense of immersion. Similarly, when there was an accident in the simulated vehicle, a smoke machine was activated for 100 ms and released a noticeable amount of smoke. After each activation of the machine, fans automatically extracted the smoke from the simulation enclosure to avoid respiratory problems.

At this point, it is important to highlight the role of user participation in the system. On the one hand, the user can be considered active when it comes to responding to the questions posed through tablets and, depending on their response, they would receive, or not, certain stimuli. On the other hand, when it comes to the film itself and the accelerations of the motion platform, the end-user is completely passive since his actions do not modify the content.

### 3.4. Gravito-Inertial Cues and Film Features

The users in the theater were placed on their seats above the motion platform as the robotic mechanism moved according to the commands sent by the simulation software. To create suitable motion cues consistent with the content of the film, this film was split into 14 scenes of less than 45 s each. Sampling frequency was set at 50 Hz, as this frequency was several times higher than the expected motion frequency. Each one of these scenes was controlled by a separate file, where the desired set of DOF for the motion platform was stored and indexed at 20 ms intervals. This also helps to ensure having continuous motion on each DOF, thus enhancing the immersiveness of the user’s experience.

These files were originally generated in two ways, depending on how the corresponding visual feed was generated. Therefore, the application provides classical motion cues—generated from simulation-based animation scenes—and non-classical motion cues—coming from real video scenes:

- *Simulation-based animation scenes:* The files corresponding to the animation scenes were recorded using the classical washout algorithm. These scenes include the simulation of virtual motion, which allows using an MCA to generate classical motion cues. Despite the fact that the classical washout can be considered quite an old scheme, it is still the main reference in the MCA literature, and it is relatively easy to tune. The implementation used in our experiments follows the Reid–Nahon [14] scheme.
- *Real video scenes*: These files were generated in a semi-manual way, so that the movement of the motion platform was similar to the visual motion that the film was showing. For this, a small software was developed. This software allowed for the mapping of the movements of a joystick with 3 analog combined axes to the desired poses of the motion platform. This recording was produced in real-time while the movie was played, without storing the values in the final file. This means that the mapping of the joystick was sent to the motion platform inverse kinematics and also to a temporary memory data structure simultaneously. This data structure allowed for the loading of the motion commands and permitted reproducing the motion again while the film was visualized, later overwriting the motion commands if they were not considered satisfactory. This process was repeated until the motion files were considered acceptable.

Once the 14 motion files were recorded, two refinement processes were applied. First, the designers prepared an initial version of the motion files and then, a set of experts prepared the final version of the motion files.

With respect to the former process, a complete installation was made in the laboratory, and the designers carried out the first experiments with the whole system once the files were recorded. These files represent the movements that the designers considered individually acceptable in a per-scene basis. Then, a complete reproduction of the whole film, including the motion platform movements, was performed in order to check whether the transmitted sensation was acceptable, especially in the different transitions between the different motion files. If the result was not as expected, the files were edited using the software explained in Section 3.5. Special care was taken with the transitions. If a transition was not smooth or the motion of the two different parts was not considered consistent, the involved scenes were edited. This process was repeated until an initial satisfactory result was obtained in the opinion of the designers.

### 3.5. Motion Edition Software and Wideband Delphi Method

**Figure 10:**
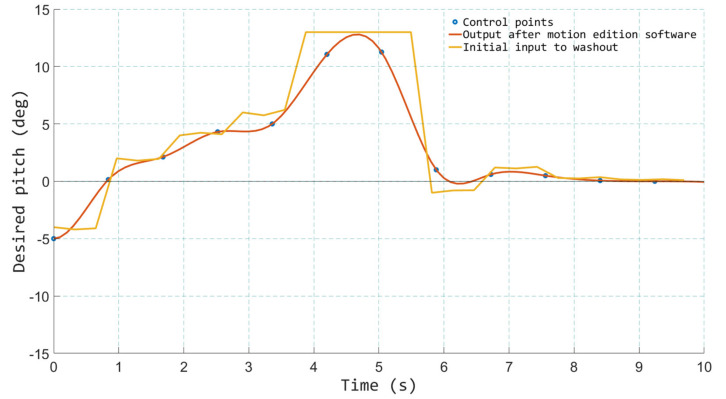
Motion edition software—modifying pitch from a saved file.

**Figure 11:**
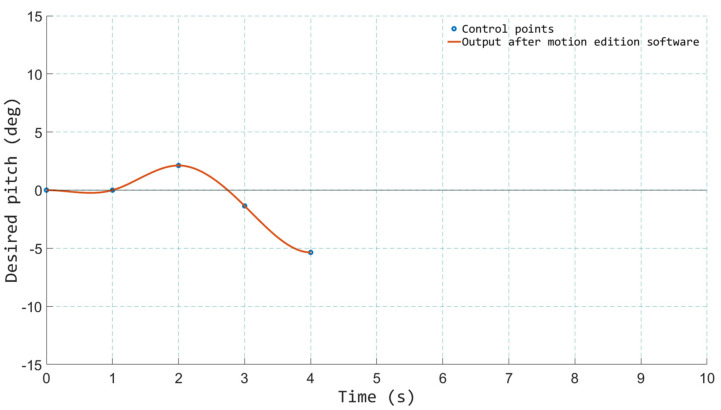
Motion edition software—creating a new motion file.

With the aim of facilitating the edition of the motion files, a software tool was created. This tool permitted the creation of new motion files, but it also allowed for the loading and manipulation of the existing ones in a very simple way. Figure 10 and Figure 11 show some screenshots of the tool.

The application allowed editing and controlling each DOF independently and also controlling all of them together so that it was possible, for instance, to scale up all the movements in case the designers wanted to transmit more abrupt sensations or scale them down in order to soften them. It was also possible to modify the time in which a certain movement was requested to the platform, advancing or delaying the motion signals. This was especially useful for adjusting the movements with what was happening at each moment in the film. In some scenes, the initial recording was performed with a joystick. Therefore, the movement of the robotic manipulator was somewhat delayed in many of the events with respect to the film, due to human reaction time and the mechanical inertia of the motion platform. This also occurs for classical motion cues generated with an MCA, in which the delay caused by the inertia and the transport delay of the motion platform cause small but noticeable desynchronizations between visual and inertial cues. This is something that cannot be fixed in real-time, and it contributes to increasing the sense of immersion in this application. As designers already know the inertia and reaction times of the robotic mechanism, they can anticipate some motion signals so that a perfect synchronization between visual and real motion is achieved.

To do this, the interface of the motion edition software allows the user to move the control points of a parametric NURBS (Non-Uniform Rational Basis Spline) curve that describes the time/position curve of each DOF. This NURBS is created from the data stored in the motion files and greatly simplifies the process of editing the motion signals. Moving the control points of the NURBS, the designers can easily make small or large corrections in the behavior of the motion platform. This tool was used in the initial phase of validation by the designers and also in the validation by experts, who were the ones who introduced the most modifications.

While carrying out the aforementioned initial setup, the hardest part was certainly tuning the motion cues of the motion platform. Thus, an expert-based refinement process was considered necessary, since the designers were not sure if the recorded motion was good enough. To this end, three experts in VR and immersive systems (with more than a decade of experience in simulators) were selected and spent three full days, alongside the designers, to validate the motion of the platform. The method used to reach an agreement on the best set of motion signals for each of the sequences was based on the Wideband Delphi technique. The modus operandi is described in Algorithm 1. The experts had access to both the parameters of the MCA and the motion edition software, so that they could rely on these two sources of information/action to tune the system.
**Algorithm 1** Delphi Wideband-Based Tuning Method*GlobalScore = 0.0* * while (GlobalScore < 8.0) do* * {* *  for each scene do* *  {* *    Play the scene with the motion platform movements* *    SceneAverageScore = 0.0* *    while (SceneAverage < 8.0) do* *    {* *      for each expert do* *      {* *        Comments for the general scene* *        {* *          Strengths* *          Weaknesses* *          Individual Cases (vehicle crash, breaking hard, etc.)* *        }* *        // 0 == movements do not correspond properly to the scene* *        // 10 == movements perfectly integrated with the scene* *        SceneScores[expert] = rated from 0 to 10* *      }* *      SceneAverageScore = SceneScore [ ] average* *      if ( SceneAverageScore < 8.0)* *        Modify movements according to the comments* *    }* *    if (scene == 1)* *      GlobalScore = SceneAverage;* *    else* *    {* *      Play the transition between each scene and the previous one* *      TransitionAverage = 0.0* *      while (TransitionAverage < 8.0) do* *      {* *        for each expert do* *        {* *          Comments for the general scene* *          TransitionScores[expert] = rated from 0 to 10* *        }* *        TransitionAverageScore = TransitionScores [ ] average* *        if (TransitionAverageScore < 8.0)* *          Modify movements according to the comments* *      }* *    }* *  }* *  Play the full film* *  for each expert do* *  {* *    FilmScore[expert] = rated from 0 to 10* *    if (FilmScore[expert] < 8.0)* *      Comment for general experience* *  }* *  GlobalScore = FilmScore [ ] average* *}*

## 4. Results and Discussion

### 4.1. Experimental Design and Procedure

Once all the files corresponding to the final tuning of the experts had been saved, the experiments with users were ready to start. Given the socio-cultural differences between the people of KSA and Spain, the authors considered that experiments with users had to be carried out with local Saudi people.

To complete the experiments, 3 groups of 12 participants were selected. Two of them were composed exclusively of men, and another one was composed exclusively of women and children because of local cultural restrictions. In the selection of users for the experiments, a wide range of ages—from 10 to 75 years—jobs (people working in offices, others in transport, etc.) and socioeconomic levels were considered.

**Figure 12:**
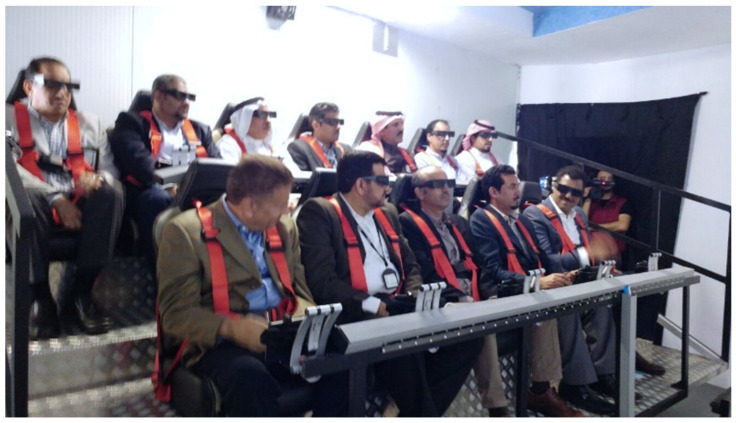
Users in the motion platform during one of the experiments.

Once the system was installed in KSA and the groups were formed, users could answer the different questions that were asked for the validation of the system. This was accomplished by means of a small application installed on the tablets that were incorporated into the theater motion structure. Figure 12 shows the experiment with one of the groups composed exclusively of men.

The experiments consisted of completing a full session with each one of the groups, playing the film before and after the modifications made by the experts in the motion signals. We played the film with the initial setup for the three groups first, and then the film with the setup validated by the experts. There was a difference of one hour between the two sessions. Therefore, the experiment analyzes and compares the initial motion setup (baseline condition) and the expert-based motion setup (final condition) using a within-subjects design.

At the end of each round, the 12 users of each group answered a series of questions about the perception they had of the movements of the motion platform. It should be considered that, given the diversity of the selected people, the questions had to be very simple to understand and answer. Table 2 describes the questionnaire that all users had to answer twice.

Once the experiments were completed by these 36 users, the system was ready to be used by all the attendees of the exhibition. A quick assessment was also performed with the general public. Users could access the exhibition facilities in groups of 50 people, and each user had to register at the entrance (check-in) and upon leaving (check-out) the exhibition, by means of tablets. Each user was given a card with a QR code that served as an identifier in the different systems of the exhibition, including the 5D theater tablet. The check-in survey requested general information about the user and a brief questionnaire regarding road safety concepts. The check-out survey had 40 questions. Some were used to see if the knowledge and awareness about road safety had improved with the exhibition. Others were used by the authors of each system in the exhibition to have a quick global assessment of the acceptance of each one of these systems by the general public. Specifically, in reference to the 5D theater, three questions were asked that can be observed in Table 3.

### 4.2. Experimental Results

One of the most critical questions to evaluate, given the profile of users targeted by the system, was whether they felt dizzy or not. Another important source of information comes from the overall rating (last question of Table 2). However, in order to get a clearer picture of the perception of the 36 test users, we created a “satisfaction ” variable that summarized the answers given to the 0–10 questions of Table 2. This satisfaction variable was a weighted average of the seven 0–10 questions of this questionnaire, with the first and last question having twice the weight of the rest.

In order to analyze the data gathered from the experiments, we applied statistical tests to the datasets. SPSS 26 was used to perform these analyses. First, it is important to test if the datasets follow a normal distribution. The Kolmogorov–Smirnov test, Anderson–Darling test and the Shapiro–Wilk test indicated that all datasets of length 36 followed a normal distribution, and therefore we can use parametric tests for paired data [39] to analyze these datasets. For the sake of brevity, we show the results only for the satisfaction variable: Kolmogorov–Smirnov D = 0.15451 and *p*-value = 0.5610; Anderson–Darling A = 0.25912 and *p*-value = 0.1209; Shapiro–Wilk W = 0.89542 and *p*-value = 0.3125.

The results of applying a paired t-test to these datasets are summarized in Table 4. As can be seen, almost all variables—six out of eight—report statistically better values in the post-test (final condition) with respect to the pre-test (baseline condition). For instance, the satisfaction variable, which summarizes the experiment, improves from 7.556 to 8.727, with a t-value of −7.146 and a *p*-value smaller than 10^−3^. Another important change occurs in the value of the general perception of the simulator, which increases from 8.361 to 9.639, an important increase. Dizziness is reduced from 2.722 to 0.306. The perception of acceleration was improved from pre-test to post-test, although its values are far from 10. The perception of turns seems to slightly increase but without a statistically significant result. The perception of the accidents also increased slightly after the manual modifications, since it goes from 6.306 to 6.528. These values are also far from 10 since the high accelerations of a collision are impossible to reproduce with a motion platform; in fact, it is not advisable at all to reproduce them even if it were possible. Finally, when asked in the pre-test if they would recommend the simulator to friends, we found that 86.1% of the users would do it, but this value changes to 100% in the post-test, even considering that some people had felt slightly dizzy. Table 5 depicts all the results averaged by groups, where variables 7 and 8 (yes/no questions) have been mapped to the 0–1 range.

In addition, the pre-test shows 13 users above the average of dizziness, fixed at a score of 2.722, with seven of them above the standard deviation. However, in the post-test, only five users above the average felt dizzy, fixed at a score of 0.306. To this, we can add that almost all the people who are rated above average, both in the pre-test and in the post-test, are framed in the group of women and juniors. On the other hand, it should be noted that, of the five users who had previously tried a simulator, only one of them showed signs of dizziness in the pre-test. This sign disappeared in the post-test.

Given the consistency of the data, we can safely deduce that the improvements are caused by the modifications introduced by the experts. This proves that the final condition is an improved condition with respect to the baseline, and thus, we can conclude that the proposed method can be useful. 

Finally, and as for the results of the three questions asked from the almost ten thousand users who have passed through the theater, 98% of users consider it correct to have incorporated a motion platform. Only 2.5% of them felt dizzy (we do not know to what degree because it is a yes/no question). The average overall rating is around 9 points out of 10.

## 5. Conclusions

After an exhaustive analysis of the obtained results, we can draw several conclusions. They can be divided into two blocks: experts and regular users. First, we will analyze the most significant comments made by the experts who performed the parameterization of the motion platform movements. Later, the conclusions about the validity of such parameterization will be drawn based on the results provided by the test users.

The first issue noted by the experts was that the movements generated by the MCA were, in many cases, too abrupt or too soft. There was no balance between them during the film. The solution to this problem was achieved by further limiting the parameters of the low-pass and high-pass filters of the MCA configuration and rebuilding the files. By doing this, the motion platform was prevented from attempting to reproduce very low or very high-frequency accelerations, leaving only the mid-frequency ones.

On the other hand, and in contradiction with what they had previously commented, experts considered that it would be interesting, from a pedagogical and simulation point of view, to emphasize certain events with specific movements of the motion platform. To be able to solve this issue—since it is not a real-time method—we put a temporary marker in each one of the scenes. With this, we had an accurate reference of the moment when we had to introduce a particular event. Specifically, we were asked to make modifications in the three collisions that were produced throughout the film, all of them placed in scenes generated from animations. Having bounded both the low-pass and high-pass filters, the impact of the vehicle went almost unnoticed.

This modification was done by adding several control points to the corresponding time zone in the pitch and roll graphs so that it could be possible to generate a momentary high-frequency movement.

On the other hand, the experts agreed that transitions from one scene to another should be as smooth as possible, preventing, for instance, a scene from ending with 10° roll and the next one from beginning with a pitch of −10°. This is something that happens recurrently since there were many changes between virtual scenes and real scenes. According to experts, those jumps between scenes would cause dizziness to users.

After the tests were performed with the three groups of users, it can be concluded that, in general, people prefer the motion of the robotic platform after the adjustments made by the experts. A clear example of this is the significant decline that occurred in people who were dizzy in the simulator. It is worth noting that Hancock [40] establishes that, in general, women are susceptible to dizziness in simulators. This is in line with the results of the first test (the original movements of the designers), but not with the results of the second one (with the movements refined by the experts), where the decline in dizziness in women has been much more significant than in men.

### Practical Implications and Future Work

Likewise, thanks to the modifications introduced by experts, almost all users agree that their perception of accidents is much more shocking than in the first tests. This is very important from the educational point of view of the system.

Overall, and in consideration of the results and shortcomings of this study, we can conclude that MCA generally works well and might have relevant applications for vehicle automation and improvements in users’ training, but there are times where their use should be restricted or modified, either because their inputs cannot be obtained, or because it is very important to reinforce certain movements, reducing others. This set of outcomes invites to keep polishing such technologies through further pilot applications and research on their ergonomics, safety and feasibility in real on-road scenarios.

**Figure 13:**
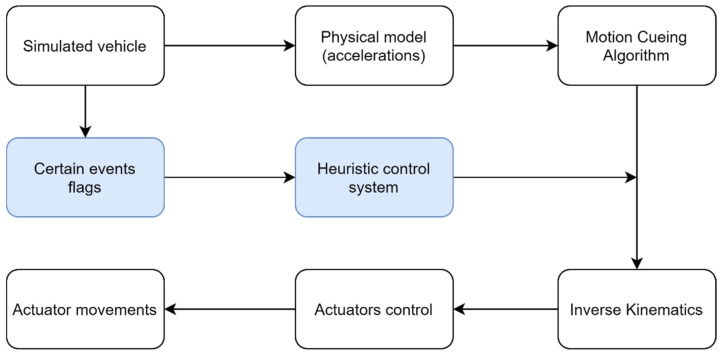
Proposed washout scheme with heuristic event control.

In addition, regarding future works, it would be very interesting to test the generation of certain heuristics for simulators in real-time that, bypassing the MCA, could be able to introduce certain movements directly to the motion platform. Of course, these heuristics must be customizable according to the target audience: the motion platform should behave differently if the user is an expert racing driver training with a complex simulator or if the user is just a little child playing a game. Figure 13 shows the scheme proposed on the basis of the results of this study. 

Another aspect that may be interesting to evaluate in the future is to study the system on categories of specific subjects, such as the elderly, the blind, or the deaf. Finally, a possible future work could include assessing and comparing classical versus non-classical motion cues.

## Figures and Tables

**Table 1:** Main features of the 3-DOF motion platform.

Motion	Range	Max. Acceleration
Pitch	±12.89°	400 °/s^2^
Roll	±10.83°	400 °/s^2^
Heave	±12.5 cm	0.5 g ≈ 5 m/s^2^

**Table 2:** The questionnaire used in the experiments.

#	Question	Response Criteria
1	Did you feel dizziness?	0 = none
		10 = yes, a lot
2	Have you been able to distinguish between a slow and a hard acceleration?	0 = not at all
		10 = yes, perfectly
3	Were you able to tell whether the vehicle was turning right or left with your eyes closed?	0 = not at all
		10 = yes, perfectly
4	Have you felt altered during the three collisions?	0 = not at all
		10 = yes, a lot
5	Do you think that the movements of the platform are suitable for all audiences?	0 = not at all
		10 = yes
6	Rate from 0 to 10 whether the motion platform has provided you with extra sensations.	0 = not at all
		10 = many
7	Have you previously experienced a simulator?	Yes
		No
8	Would you recommend this experience to your friends?	Yes
		No
9	Rate the general experience from 0 to 10.	0 = very bad
		10 = very good

**Table 3:** Relevant questions in the check-out survey.

#	Question	Response Criteria
1	Did you find it appropriate to incorporate motion in the 5D cinema in order to provide it with more realism?	Yes
		No
2	Did you feel dizzy in the 5D cinema?	Yes
		No
3	Overall rating of 5D cinema from 0 to 10.	0 = very poor
		10 = very good

**Table 4:** Results of the statistical tests applied to the datasets of length 36.

Question/ Variable	Baseline (Pre-Test)		Final (Post-Test)		t-Value	*p*-Value
	Mean	Standard Deviation	Mean	Standard Deviation		
1	2.722	2.972	0.306	0.856	5.506	<10^−3^
2	6.361	1.988	7.417	2.048	–5.012	<10^−3^
3	7.056	2.042	7.333	1.957	–1.616	0.115
4	6.306	2.713	6.528	2.823	–2.092	0.044
5	8.111	1.894	9.694	0.951	–5.202	<10^−3^
6	9.167	1.082	9.389	0.964	–1.348	0.186
9	8.361	1.839	9.639	0.867	–4.154	<10^−3^
Satisfaction	7.556	1.234	8.727	0.700	–7.146	<10^−3^
Degrees of freedom = 35						

**Table 5:** Results averaged by gender-based groups.

Question/ Variable	Women			Men 1			Men 2		
	Pre-Test	Post-Test	% Diff	Pre-Test	Post-Test	% Diff	Pre-Test	Post-Test	% Diff
1	4.25	0.67	−84.3%	1.50	0.08	−94.4%	2.42	0.17	−93.1%
2	6.08	7.00	15.1%	6.67	7.67	15.0%	6.33	7.58	19.7%
3	5.67	6.42	13.2%	7.75	7.83	1.1%	7.75	7.75	0.0%
4	8.33	8.67	4.0%	5.33	5.50	3.1%	5.25	5.42	3.2%
5	6.58	9.33	41.8%	9.00	9.92	10.2%	8.75	9.83	12.4%
6	9.33	9.33	0.0%	9.58	9.58	0.0%	9.83	9.83	0.0%
7	0.00	0.00	–	0.17	0.17	0.0%	0.25	0.25	0.0%
8	0.75	1.00	33.3%	0.92	1.00	9.1%	0.92	1.00	9.1%
9	7.67	9.58	25.0%	8.42	9.75	15.8%	9.00	9.58	6.5%
Satisfaction	6.98	8.69	24.5%	7.91	8.79	11.1%	7.78	8.71	11.9%

## Data Availability

The study data can be accessible upon reasonable request to the corresponding author.
